# Targeting Neutrophil Extracellular Trap Formation: Exploring Promising Pharmacological Strategies for the Treatment of Preeclampsia

**DOI:** 10.3390/ph17050605

**Published:** 2024-05-09

**Authors:** Leticia Lorena Hernández González, Laura Pérez-Campos Mayoral, María Teresa Hernández-Huerta, Gabriel Mayoral Andrade, Margarito Martínez Cruz, Edgar Ramos-Martínez, Eduardo Pérez-Campos Mayoral, Víctor Cruz Hernández, Ismael Antonio García, Carlos Alberto Matias-Cervantes, Miriam Emily Avendaño Villegas, Carlos Mauricio Lastre Domínguez, Carlos Romero Díaz, Juan de Dios Ruiz-Rosado, Eduardo Pérez-Campos

**Affiliations:** 1 National Technology of Mexico/IT Oaxaca, Oaxaca de Juárez, Oaxaca 68030, Mexico; leticialorenahg@gmail.com (L.L.H.G.); mcruz@itoaxaca.edu.mx (M.M.C.); carlos.lastre@itoaxaca.edu.mx (C.M.L.D.); carlosrom74@hotmail.com (C.R.D.); 2 Faculty of Biological Systems and Technological Innovation, Autonomous University “Benito Juárez” of Oaxaca, Oaxaca 68125, Mexico; 3 Research Center, Faculty of Medicine UNAM-UABJO, Autonomous University “Benito Juárez” of Oaxaca (UABJO), Oaxaca 68020, Mexico; lperezcampos.fmc@uabjo.mx (L.P.-C.M.); gmayoral.fmc@uabjo.mx (G.M.A.); eperezcampos.fmc@uabjo.mx (E.P.-C.M.); 4 CONAHCyT, Faculty of Medicine and Surgery, Autonomous University “Benito Juárez” of Oaxaca (UABJO), Oaxaca 68020, Mexico; mthernandez@conahcyt.mx (M.T.H.-H.); cmatias@conahcyt.mx (C.A.M.-C.); 5 School of Sciences, Autonomous University “Benito Juárez” of Oaxaca (UABJO), Oaxaca 68020, Mexico; eramos.ciencias@uabjo.mx; 6 “Dr Aurelio Valdivieso” Hospital General. SS., Oaxaca 68040, Mexicois_angar6@yahoo.com (I.A.G.); 7 Kidney and Urinary Tract Research Center, Abigail Wexner Research Institute, Nationwide Children’s Hospital, Columbus, OH 43215, USA; 8 Division of Nephrology and Hypertension, Nationwide Children’s Hospital, Columbus, OH 43205, USA; 9 Clinical Pathology Laboratory, “Eduardo Pérez Ortega”, Oaxaca 68000, Mexico

**Keywords:** neutrophil extracellular traps, NETs, preeclampsia, pregnancy, neutrophils, oxidative stress, hypertension, NF-κβ inhibitors, vitamin D, aspirin

## Abstract

Neutrophils, which constitute the most abundant leukocytes in human blood, emerge as crucial players in the induction of endothelial cell death and the modulation of endothelial cell responses under both physiological and pathological conditions. The hallmark of preeclampsia is endothelial dysfunction induced by systemic inflammation, in which neutrophils, particularly through the formation of neutrophil extracellular traps (NETs), play a pivotal role in the development and perpetuation of endothelial dysfunction and the hypertensive state. Considering the potential of numerous pharmaceutical agents to attenuate NET formation (NETosis) in preeclampsia, a comprehensive assessment of the extensively studied candidates becomes imperative. This review aims to identify mechanisms associated with the induction and negative regulation of NETs in the context of preeclampsia. We discuss potential drugs to modulate NETosis, such as NF-κβ inhibitors, vitamin D, and aspirin, and their association with mutagenicity and genotoxicity. Strong evidence supports the notion that molecules involved in the activation of NETs could serve as promising targets for the treatment of preeclampsia.

## 1. Introduction

Preeclampsia (PE) is a multifactorial and multisystem syndrome specific to human pregnancy [1]. It manifests as hypertension that emerges at 20 weeks of gestation in previously normotensive women [2,3]. 

PE is categorized into subtypes, including early and late-onset, as well as severe, mild, and mild to severe [4]. From a haemodynamic perspective, based on the value of cardiac output (CO) at rest, PE is divided into two types, hypo- or hypervolemic. The hypovolemic type also denoted as “classic” or placental early onset, is characterized by placental hypertension induced by vasoconstriction [5]. It presents inadequate placental perfusion, endothelial damage, placental insufficiency, and elevated levels of anti-angiogenic molecules such as soluble fms-like tyrosine kinase 1 (sFlt-1) [6] and soluble endoglin (sEng). The hypervolemic, maternal, or term type is more prevalent. This variant is associated with elevated CO, increased oedema due to water retention, augmented placental perfusion, higher foetal birth weight, relaxed vasculature, and maternal obesity [7]. 

Severe preeclampsia can lead to the development of HELLP syndrome (Haemolysis, Elevated Liver enzyme, and Low Platelets). This syndrome is characterised by microangiopathic haemolytic anaemia, hypertension, proteinuria, oedema, liver dysfunction, thrombocytopenia [8], increased CO, and inotropy [9]. 

Furthermore, endothelial cells, platelets, and neutrophils play pivotal roles in the inflammatory, hypertensive, and prothrombotic processes observed in preeclampsia [10,11,12]. Although distinct neutrophil subpopulations have been characterized in different human pathologies and experimental models [13,14,15,16,17,18], their specific roles in preeclampsia remain under investigation. Numerous groups of molecules orchestrate the relationships between neutrophils, platelets, and endothelial cells, such as the P-selectin-PSGL-1 axis [19] via phosphodiesterase type-4 or Src family kinases and the vWF-glycoprotein Ibα axis [20].

Studies have demonstrated alterations in the quantity and functionality of phagocytosis, degranulation, extracellular vesicle release, and the release of neutrophil extracellular traps (NETs) in preeclampsia. These changes are intricately linked to the systemic inflammatory and immune state. Furthermore, there is compelling evidence indicating increased elastase [21] and nucleosomes [22] release, particularly in early-onset PE. These findings suggest that alterations in neutrophil functions, such as NETosis, may contribute to inflammation and endothelial dysfunction, thereby promoting the development of PE.

In this review, we discuss the role of neutrophil activation and NETosis in the context of preeclampsia. Additionally, we examined pharmacological compounds that exhibit a negative regulatory influence on NETs, which could potentially be employed in the treatment of preeclampsia. Additionally, we explored the mutagenicity and genotoxicity associated with the use of these compounds.

To complete this review, we conducted a focused search and discussed the selection of drugs for analysis with a panel of experts using a Delphi exercise. We searched on PubMed, Google Scholar, and the Cochrane Library from 1 January 2023 to 31 January 2024, using the search terms “preeclampsia”, “neutrophils”, “neutrophil extracellular traps”, “NETs”, “NETosis”, “preeclampsia and oxidative stress”, and “preeclampsia and NF-κβ inhibitors”; we also cross-referenced these terms with the following: “aspirin”, “acetylsalicylic acid”, “dexamethasone”, “glucocorticoids”, “resveratrol”, “cyclosporine A”, “azithromycin”, “chloramphenicol”, “metformin”, “hydroxychloroquine”, “heparin”, “vitamin D”, “disulfiram”, “curcumin”, “roflumilast, “apremilast”, “rolipram, “*Glycyrrhiza glabra*”, “activated protein C”, and “recombinant human DNase I”.

## 2. Activation of Neutrophils in PE

Neutrophil activation in preeclampsia is characterized by distinct molecular and functional changes. An analysis of the expression of 40 inflammation-related genes in leukocytes has revealed a significant increase in the mRNA expression of the nuclear factor of Kappa light chain (NFKβ 1A), Cyclin-dependent kinase inhibitor 1A (CDKN-1A), Interleukin-8 (IL)-8, and IL-1b genes in leukocytes of pregnant women with PE compared to their healthy counterparts. This heightened expression favours the polarization of neutrophils towards an inflammatory profile [23]. Additionally, peripheral blood neutrophils in PE show lower expression of apoptosis markers compared to neutrophils from healthy pregnant women. Higher expression of these markers of apoptosis was observed in neutrophils from non-pregnant women, suggesting that delayed neutrophil apoptosis contributes to complications during pregnancy, such as PE [24,25].

In PE, neutrophil granule release and ROS production cause direct damage to the cell membrane through peroxidation of membrane lipids, leading to cell death [26]. This phenomenon triggers endothelial activation characterized by an increased expression of vascular cell adhesion molecule-1 (VCAM-1) and inactivation of factors such as endothelium-derived relaxing and prostacyclin (PGI2). In addition, there is an increase in the number of activated neutrophils, which is associated with a programmed neutrophil extracellular traps formation (NETosis) [27,28,29].

Neutrophils from pregnant women with PE exhibit increased expression of the adhesion molecule CD11b, which serves as a marker of cell activation [30]. Furthermore, there is a notable reduction in the surface expression of L-selectin (CD62L) on CD15+ neutrophils, which correlates with immune activation in PE [31]. Studies have shown that NETs can induce platelet activation and a prothrombotic state during PE [28,32]. This body of evidence suggests that neutrophils are activated and potentially primed for NET release in PE, both in the microcirculation and periphery, contributing to a feedback loop involving inflammation and endothelial dysfunction. 

## 3. Neutrophils in the Uteroplacental Microvasculature in PE

In the uteroplacental microvasculature of pregnant women with PE, there is a marked infiltration of neutrophils associated with increased expression of the cell adhesion molecule ICAM-1 and a positive gradient of IL-8. In addition, expression of the β-2 integrins CD11a, CD11b, and CD11c is increased in neutrophils from women with PE compared to their healthy counterparts [33]. This heightened presence of neutrophils and associated molecules in the maternal tissue vasculature can cause vasoconstriction, ischemia, oxidative stress, inflammation, and subsequent endothelial dysfunction [34]. 

Analyses of biopsies obtained by caesarean section in pregnant women with PE have shown that the number of neutrophils attached to the vasculature is elevated [35,36]. A higher concentration of neutrophils in the vasculature potentially contributes to endothelial dysfunction [37].

The syncytiotrophoblast, the membrane layer that covers the placenta and is in direct contact with maternal blood, releases microvesicles (placental syncytiotrophoblast microvesicles, STBMs) [38] that can stimulate ROS production in neutrophils [39]. Syncytiotrophoblast cells and serum from pregnant women with PE contain higher levels of IL-32β compared to healthy pregnant women. IL-32 exerts multiple functions such as NET induction [40]. 

## 4. NETs

NETs are structures consisting of nuclear or mitochondrial DNA with proteins such as lactoferrin, actin, histones, neutrophil elastase (NE), and myeloperoxidase (MPO) [41]. The main function of NETs is the containment and elimination of pathogens [42,43]; however, their participation in thrombosis, autoimmune diseases, cancer, diabetes [44], and cardiovascular diseases such as hypertension [45] has also been reported.

NETosis can be triggered by various stimuli, including microbial pathogens, inflammatory cytokines, and chemical agents. The formation of NETs relies on histone citrullination, catalysed by elevated levels of neutrophil peptidyl-arginine deaminase-4 (PAD4). Additionally, NET formation depends on the production of ROS and activation of MPO and NE [46], leading to nucleosome histone digestion, chromatin decondensation, and release of DNA and antimicrobial molecules [47]. 

**Figure 1:**
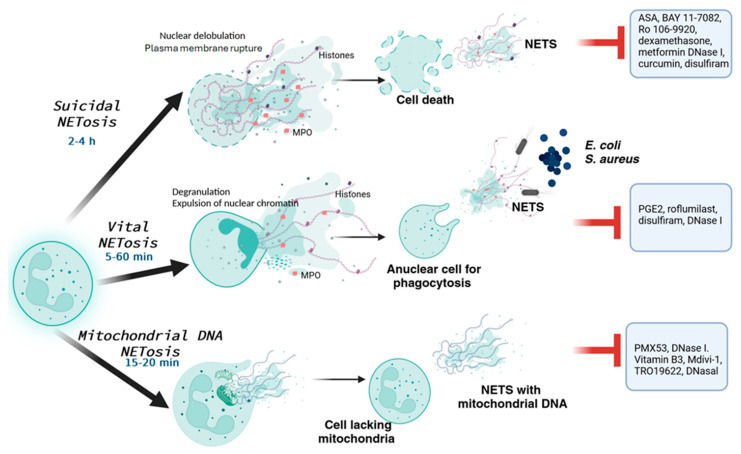
Mechanisms of NET formation. Neutrophils generate NETs in response to various stimuli. Four types of NETs have been described based on structural changes during their formation. These changes involve the activation of different signalling pathways. In the case of mitochondrial NETosis, nuclear DNA remains inside the cell and has been observed in specific cases.

There are several signalling pathways involved in NET formation [48]; one pathway involves the generation of ROS through the nicotinamide adenine dinucleotide phosphate (NADPH) oxidase. Activation of the NADPH oxidase complex 2 (NOX2) triggers the Raf/MEK/ERK pathway and augments cytosolic ROS levels [49], resulting in citrullinated histone H3-dependent (Cit-Histone H3) lytic or suicidal NETosis, Figure 1. These lytic NETs are released by the presence of extracellular microbes, fungi, viruses, interferon, phorbol myristate acetate (PMA), IL-8, antibody–antigen complexes, autoantibodies, and concanavalin A.

NOX2-independent pathways can lead to the formation of non-lytic NETs, also known as vital NETs [50]. These NETs are released in the presence of *Staphylococcus aureus* (*S. aureus*), *Escherichia coli* (*E. coli*), damage-associated molecular patterns (DAMPs), platelets, Toll-like receptors 2/4 (TLR2/4), and lymphocytes associated with a function neutrophil antigen 1 (LFA1).

Other less-studied pathways that induce NETs include those involving signal inhibitory receptor 1 (SIRL1) [51] and extracellular cold-inducible RNA-binding protein (eCIRP) through PD4 [52]. In addition, the formation of mitochondria-dependent vital NETs by silent information regulator 1 (SIRT1) that has been reported in both tumour-associated aged neutrophils (Naged, CXCR4+ CD62L low) in breast cancer lung metastasis [53] and non-tumour pathologies [54] has not been studied in preeclampsia.

NETs can be identified and quantified by distinct visualisation methods or through markers of their released products. Visualisation methods use molecules that are intercalated into DNA, such as propidium iodide and SYTOX Orange. On the other hand, the markers include those that detect DNA-histone complexes (nucleosomes), MPO [55], circulating MPO complexes (MPO)-DNA [56], surface citrullinated histone (H3cit), double-stranded DNA (ds), myeloid-related protein (MRP), DNase, and elastase [57,58].

## 5. NETs in PE

In PE, there is an increased trafficking of foetal cells into the maternal circulation. The elevated concentration of DNA in blood from foetal-derived cells could activate the immune response and induce cytokine production, potentially leading to pregnancy complications and an increased risk of foetal rejection. 

Higher concentrations of NET components (DNA, histones, and MPO) have been reported in the plasma of pregnant women with preeclampsia compared to healthy pregnant women [55]. The first molecule associated with neutrophil activation in the blood of women with PE was neutrophil elastase [59].

Concordantly, in vitro experiments have shown that neutrophils from pregnant women with PE are more likely to form NETs compared to healthy pregnant and non-pregnant women [28]. Also, women with PE and obesity have a higher presence of MPO in their systemic vasculature. MPO catalyses the formation of ROS and RNS in PE by consuming circulating nitric oxide [60,61].

Marder et al. analysed placental samples from women with PE, healthy pregnant women, and abdominal tissue from non-pregnant women, revealing that MPO expression is elevated in tissues from pregnant women compared to non-pregnant women. Notably, NETs were found in the intervillous space in PE [62].

Using anti-NE, Gupta et al. demonstrated that NETs were present in the vicinity of the syncytiotrophoblastic layer in healthy pregnant and preeclamptic women. However, the intervillous space was frequently infiltrated by numerous NETs, and their presence increased in PE [63]. Anti-histone 2A (H2A) staining showed that H2A was significantly increased in the intervillous space of women with PE [64]. Gupta et al. showed that inflammatory syncytiotrophoblast microparticles (STBM) and IL-8-induced neutrophil extracellular DNA extrusion increased CD11b expression and ROS generation in PE [65].

PE is associated with markers of autoimmunity, including anti-β2 glycoprotein-I (ab2GPI); anticardiolipin antibodies (aCL); lupus anticoagulant (LA); β1, β2, and α1 adrenoreceptors; prothrombin; endothelin-1 receptor type A (ETA-AA); and Angiotensin II receptor type 1(AT1-AA) antibodies [66,67] as well as antibodies with different specificity than AT1-AA [68]. Furthermore, the level of antineutrophil cytoplasmic autoantibodies (ANCA) in PE patients was significantly elevated compared with a healthy pregnancy [69]. Although these autoantibodies may induce NETosis during PE, this hypothesis remains to be addressed.

**Figure 2:**
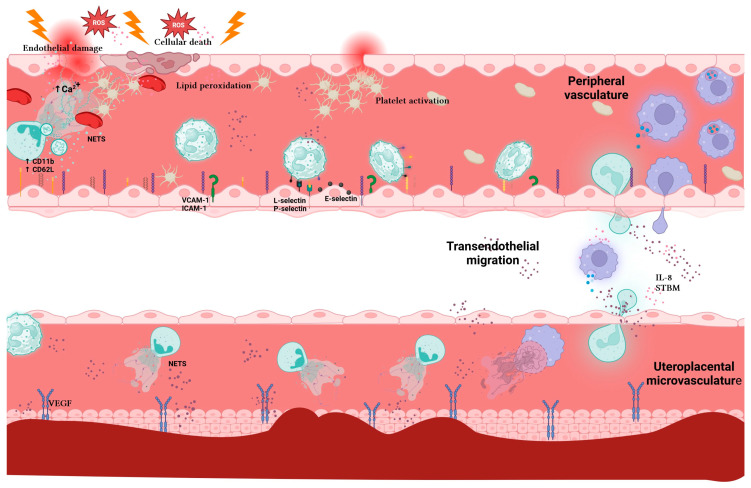
NETs in preeclampsia. In preeclampsia, neutrophils can be activated in two different environments. On the one hand, systemic inflammation with a consequent release of cytokines, DAMPs, and activation of platelets and macrophages stimulates neutrophils, promoting the expression of selectins and their migration to the site of damage, such as the uteroplacental microvasculature where NETs will be released. In addition, neutrophil activation in the peripheral vasculature can lead to the formation of NETs. These networks and their components promote thrombosis, inflammation, damage, and endothelial dysregulation. Likewise, inflammation and ischemia activate neutrophils resident in the uteroplacental tissue, promoting the formation of NETs. These NETs perpetuate and increase inflammation, damaging local tissues and promoting the formation of inflammatory mediators that reach the circulation, attracting more neutrophils.

Altogether, the studies mentioned above provide a compelling association between preeclampsia and the increased presence of NETs in pregnant women, Figure 2. Strategies targeting NETs in preeclampsia may hold promise for mitigating immune responses and complications during pregnancy.

## 6. Pharmacological Modulation of NETs

NETs can be targeted through different drugs with diverse mechanisms of action, Table 1. 

**Figure 3:**
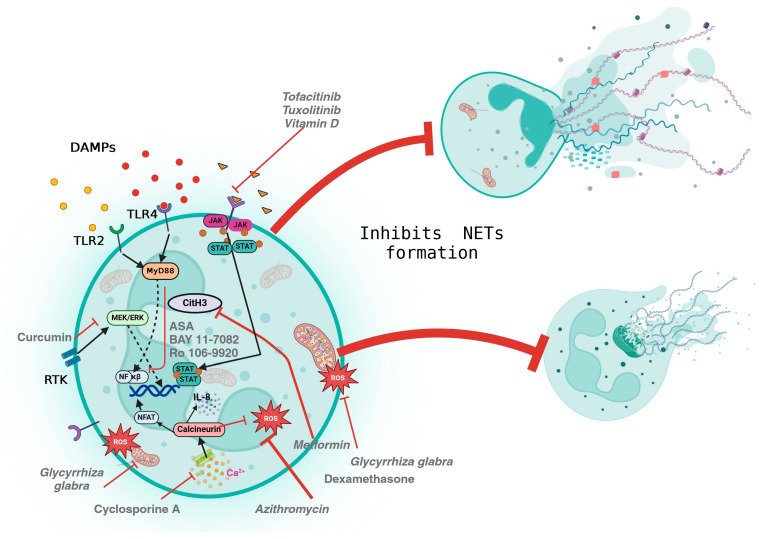
Drugs and molecules that reduce neutrophil extracellular traps (NETs). Signalling pathways involved in reducing NETs include Raf-MEK-ERK signalling, calcineurin signalling, phosphorylation of the p65 subunit of NF-κβ via Rac2, PAD4, and block receptors with monoclonal antibodies and DNAases.

Several pharmacological agents are currently being studied for the treatment of moderate and severe preeclampsia (Figure 3), including aspirin, sildenafil citrate, hydroxychloroquine, pravastatin, metformin, magnesium sulphate, nifedipine, labetalol, and nitro-glycerine. However, only one trial has been reported, on clinicaltrials.gov, that was designed to inhibit NET formation in PE using Toll-like receptor (TLR) blocking antibodies. Some agents mitigate NETs by inhibiting ROS, including flavonoids like epicatechin and rutin, along with vitamin C, 5-aminosalicylic acid (5-ASA), N-acetyl-L-cysteine (NAC), dexamethasone, azithromycin, and *Glycyrrhiza glabra* [70,71]. Others inhibit the JAK/STAT pathway such as tofacitinib, ruxolitinib, and baricitinib [72]. Furthermore, interleukin antagonists, such as tocilizumab, canakinumab, anakinra, and rilonacept, also modulate NETs [73].

Amid the COVID-19 pandemic, there has been a surge in accelerated investigations on drugs and molecules repurposed for their potential to inhibit NETs, as summarized in Table 1. Some of them have garnered attention for their well-established anti-inflammatory effects. Some have not only been utilized in inflammation management but also in aiding lung maturation during preterm births and mitigating preeclampsia [74,75,76,77,78,79,80,81]. Aspirin, vitamin D, and recombinant human DNase I have emerged as candidates worthy of study due to their reported lower risk or absence of mutagenicity and genotoxicity.

The Raf-MEK-ERK signalling pathway is involved in NET formation through the activation of NADPH oxidase. Drugs that reduce NET formation through this pathway include curcumin, GW5074 [82], and Celastrol [83]. While drugs that inhibit NETs by inhibiting the phosphorylation of the p65 subunit of NF-κβ are anti-inflammatory drugs such as acetylsalicylic acid (ASA), BAY 11-7082, and Ro 106-9920 [84].

On the other hand, cyclosporine A uses the calcineurin pathway to reduce NETs [85], while the antioxidants N-acetylcysteine, ethotrexate, trolox, tempol [86], epigallocatechin-3-gallate [87], and diphenyleneiodonium chloride (DPI) [88] decrease NETs by reducing mitochondrial ROS formation [85]. DPI also reduces NET formation through the PKC-βII pathway [89] like metformin. Other drugs that have been shown to significantly inhibit ROS-dependent NET production are propofol and lipid emulsion [90].

Furthermore, drugs such as Hydroxychloroquine (HCQ) [91], PF3758309, and IPA-3 inhibit NETs through Rac2 [92]. Chloroquine (CQ) and HCQ can prevent NET formation by inhibiting PAD4 [93] or TLR signalling as with HCQ, CQ, and enpatoran [94]. In addition, PDE4 inhibitors such as apremilast, rolipram, and crisaborole can reduce the formation of NETs [95]. 

**Table 1:** Molecules that downregulate NETosis.

Drugs Substance	Mechanism of Action	Evaluated for NOX-Dependent (NOX-D) and ROS-Independent (ROX-I) NET Formation	Clinical Trials/Models/Examples	Mutagenicity and Genotoxicity In Vitro
**Drugs**				
Aspirin (ASA)	Human neutrophils were stimulated with Phorbol 12-myristate 13-acetate (PMA) or TNF-α. In addition, ASA, BAY 11-7082, and Ro 106-9920 prevented the formation of NETs by reducing the phosphorylation of the p65 subunit of NF-κβ [84].	**NOX-D**	In isolated neutrophils stimulated with sodium hydroxide, ASA can enhance the migration of corneal epithelial cells (HCEs) and reduce the formation of NETs [96].	ASA can protect against genotoxicity [97].
Dexamethasone	Inhibits neutrophil functions such as intracellular ROS, degranulation, and NETosis.	**NOX-D**	Neutrophils cultured with dexamethasone showed reduced NET formation, after stimulation with PMA [98].	It can induce significant DNA damage in human cells; however, it passes the Ames/*Salmonella* assay [99,100].
Resveratrol	Decreases the release of free DNA from neutrophils and NETosis	**NOX-D**	During in vitro tests in the presence of PMA, it was shown that resveratrol decreases the formation of NETs and cytokine production in healthy controls and with COVID-19 [101].	Despite its genotoxic effects, it does not cause mutagenesis and is used for its genotoxic activity against gastric cell adenocarcinoma [102,103].
Cyclosporine A	Inhibits IL-8-induced NETosis by inhibiting the calcineurin pathway.	**NOX-D**	In isolated neutrophils, stimulated with PMA or ionomycin, treated with Cyclosporine A or ascomycin, the formation of NETs decreases [104].	It is not genotoxic in humans. It inhibits the protein phosphatase calcineurin and can induce lymphoma in Xpa/p53 mice [105].
Azithromycin	Decreases the production of ROS	**NOX-D**	Pre-treatment with Azithromycin decreases NETosis in neutrophils isolated from PMA-stimulated healthy subjects. This effect is observed at low doses [106].	It does not induce mutations or chromosomal aberrations in microbial or mammalian cells [107].
Chloramphenicol	Reduces the formation of NETs, possibly by inhibiting myeloperoxidase (MPO)	**NOX-D**	Pre-treatment with chloramphenicol reduces PMA-induced NET release [94].	In rodents and human cells, it is a pro-mutagenic compound [108].
Metformin	Affects nuclear dynamics (delobulation and decondensation) as well as PKC-βII membrane translocation and NADPH oxidase activation in neutrophils [109].	**NOX-D**	Metformin decreases NETosis and its components such as elastase, proteinase-3, histones, and double-strand DNA in PMA-stimulated neutrophils in in vitro and clinical trial samples [109].	There is conflicting evidence about the effects of metformin. Micronucleus assay suggests it may be genotoxic; however, analyses using chromosomal aberration (CA) and cytokinesis-block micronucleus (CBMN) assay report that it has a radioprotective effect on DNA damage and apoptosis in human lymphocytes [110,111].
Hydroxychloroquine	Inhibits the expression of PAC4, Rac2, and the formation of NETs	**NOX-D**	Hydroxychloroquine alleviates hepatic ischemia/reperfusion (IR) injury in severe combined immunodeficiency (SCID) mice and C57BL/6 mice by inhibiting NETosis [91].	It induces both oxidative DNA damage detected by 8-oxodG and the induction of mutants in mouse embryonic fibroblasts [112].
Heparin	NET molecules, such as neutrophil elastase, interact with heparin and heparin oligomers to form molecular complexes that can regulate NETosis [113]. Low molecular weight heparin (LMWH) can repair nucleosome/histone 3-mediated damage in trophoblasts [69].	**--**	In vitro studies have shown that heparinized adsorbents such as heparin sepharose deplete PF4, histones/nucleosomes, and HMGB1 [114]. Heparin pre-treatment decreased serum and lung NETs in a C57BL/6J mice model [115]. Circulating histones bound to H3 and H4 nucleosomes are increased in patients with preeclampsia and intrauterine growth restriction. H3 affects extravillous trophoblast migration, invasion, and survival. This effect can be reversed in vitro by LMWH, but not with ASA [69].	LMWH does not show any mutagenic activity [116,117].
Vitamin D	Vitamin D supplementation has been shown to reduce the risk of preeclampsia [118], as well as decrease the generation of NETs, particularly when combined with omega-3 PUFAs [119].	**NOX-D**	PMA-stimulated neutrophils from patients with systemic lupus erythematosus (SLE) and hypovitaminosis D were treated with calcitriol/1,25(OH)_2_D3. The authors reported a dose-independent decrease in externalised neutrophil elastase (NE) during NETosis [120].	In cancer rodents treated with cyclophosphamide, it reduced the frequency of chromosomal aberrations in Chinese hamster lung cells and reduced micronuclei and lymphocyte damage in mice [121].
Disulfiram	Inhibits NETs [122].	**NOX-D**	It reduced NETs and perivascular fibrosis and downregulated innate immune and complement/coagulation pathways [111].	--
Curcumin	Inhibits the generation of NETs by suppressing the MEK/ERK pathway [123].	**NOX-D**	In a mouse model, curcumin was shown to reduce hepatic ischemia-reperfusion injury by inhibiting NET formation [91].	High concentrations are cytotoxic and increase the frequency of micronuclei in PC12 cells; at low doses, it reduces the number of micronuclei induced by cisplatin [124].
Phosphodiesterase Type-4 (PDE4) inhibitors such as Roflumilast (Daliresp), Apremilast (Otezla), and Rolipram	Roflumilast blocks PDE4 and reduces in vitro and in vivo NETosis in animal models [125]. Inhibition of PDE4 by rolipram prevents the adhesion of platelets and neutrophils, which involves members of the Src family kinase (SFK) [126,127].	**ROX-I**	Clinical trials have been conducted with Roflumilast for severe chronic obstructive pulmonary disease (COPD) and with Otezla for psoriasis [128].	--
*Glycyrrhiza glabra*	Inhibits ROS, mitochondrial ROS (mtROS), NET generation, and cytokine release.	**--**	In an animal model, *Glycyrrhiza glabra* was proven to decrease COVID-19 pathology by reducing NETosis [129].	--
**Protein molecules**				
Activated protein C (APC)	Cleaves and detoxifies extracellular histones and its effect on reducing NETs dependent on endothelial protein C receptor (EPCR), protease-activated receptor 3 (PAR3), and macrophage antigen-1 (Mac-1) [130].	**--**	A clinical trial was conducted evaluating the safety and efficacy of recombinant human-activated protein C (rhAPC; drotrecogin alfa [activated]) in preeclampsia, but further studies are needed [131]. APC variants have been designed to have a greater ability to destroy histone H3 with fewer anticoagulant properties [132].	Drotrecogin alfa (activated) has not been studied for carcinogenicity [133].
Recombinant human DNase I (rhDNase I, rhDNase, Pulmozyme^®^, dornase alfa)	Hydrolyses extracellular DNA released by neutrophils [134].	**--**	Intravenous administration of rhDNase to mice degraded NETs and attenuated coagulopathy in the acute respiratory distress syndrome (ARDS) model [135].	It does not show cytotoxicity in human peripheral blood mononuclear cells [136].

Other molecules such as PGE2, which has been used in labour induction, decrease NET formation through cAMP production. However, there are also no reports of trials with PGE2 to reduce NETs in preeclampsia [137,138,139].

Others, although they reduce NET formation, are not considered due to the risk of mutagenicity or genotoxicity during pregnancy. A mutagenic agent causes damage to DNA and results in mutation, while a genotoxic agent can cause damage to DNA or chromosomes but without necessarily resulting in mutation [140], such as 5-fluorouracil (5FU) [141].

## 7. Therapeutic Approaches to Inhibit NET Formation in PE

Low-dose aspirin (75 mg) is used in the clinic to prevent preeclampsia; its best-known action is the inhibition of prostaglandin and thromboxane A2 synthesis by cyclooxygenases COX-1 and COX-2. 

Preeclampsia is characterized by elevation of TXA2 and decreased prostacyclin. TXA2 is a procoagulant molecule related to thrombosis observed in preeclampsia; prostacyclin PG1 is a vasodilator molecule. In PE, there is an increase in TXA and a decrease in PGI; aspirin acts on both, although platelets are the main producers of TXA2 [142]. 

PE is related to an inflammatory and prothrombotic state, and thus the effect of aspirin in inhibiting inflammation and platelet aggregation is considered to prevent and treat it; however, beyond its antithrombotic action, the effect of aspirin on the activation and modulation of the inflammatory response of neutrophils has recently been studied.

During pregnancy, neutrophils express PAR-1 and F2R, which are thrombin receptors. Aspirin prevented p65 translocation of NF-kβ to the nucleus and TXA2 production in neutrophils from pregnant women via PAR-1. In addition, aspirin inhibits lipid peroxidation by COX-2 in pregnant neutrophils [143]. These PAR-1 and F2R receptors are not expressed in neutrophils from pregnant women.

Both aspirin and glucocorticoids reduce NF-κβ activity. It is a family of transcription factors, phosphorylated p65 (RelA), RelB, c-Rel, p105/p50 (NF-κβ1) and p100/52 (NF-κβ2), that regulate the immune response, inflammatory response, apoptosis and proliferation [144].

In PE, neutrophil adhesion to the vasculature increases. This adhesion could be reduced in the presence of an intermediate in aspirin metabolism and aspirin-activated lipoxin A4 (ATL, 15-epi-LXA4) [145]. Aspirin generally inhibits NETosis, but more studies are needed to analyse its usefulness in preeclampsia. 

Some meta-analyses and individual cases have shown that low-dose aspirin can prevent preeclampsia in people with a risk factor, but aspirin crosses the placental barrier and can inhibit foetal platelet aggregation, increasing the risk of spontaneous abortion; thus, its use is under medical supervision. The use of aspirin has been empirical, but more data are needed to implement it as a treatment [146].

Furthermore, the therapeutic use of glucocorticoids in PE is primarily indicated in cases of foetal growth restriction. It has been proposed that single doses after week 24 (2 doses of 12 mg betamethasone 24 h apart or 6 mg dexamethasone 12 h apart before delivery) help foetal lung maturation in PE [147,148]. Dexamethasone has been shown to transiently restore absent end-diastolic flow in the umbilical artery in early-onset preeclampsia [149]. However, dexamethasone treatment for PE should be carefully considered, as the use of high doses and repeated doses should be avoided for fear of possible long-term adverse effects on the foetal brain [150].

On the other hand, glucocorticoids such as dexamethasone, prednisone, and rimexolone interact through glucocorticoid receptor antagonism in PE [151]. Glucocorticoid receptors are expressed in the trophoblast, and their inhibition favours the expression of angiotensin 2 receptor antagonist (ART2), which has vasodilatory effects. In normal pregnancy, ART2 predominates, reducing hypertension, while ART1, which has a vasoconstrictor effect, increases in PE [152]. Numerous studies show that dexamethasone in early pregnancy has harmful effects such as deficient trophoblast development, increased trophoblast invasion inhibitor SERPINE1, and increased systolic blood pressure [153,154], even causing DNA damage (Table 1). Dexamethasone caused abnormal mitochondrial morphology and mitochondrial dysfunction in the placentas of pregnant rats, in addition to altering placental signalling pathways such as oxidative phosphorylation, inflammation, and the insulin-like growth factor system [155].

PE is characterized by a state of systemic inflammation where platelet abnormalities occur. It has been shown that dexamethasone significantly inhibited degranulation, intracellular ROS production, CXCL8 release, and neutrophil NETosis in patients with severe COVID-19 pneumonia [98]. Dexamethasone has been shown to inhibit *Staphylococcus aureus*-induced NET formation via upregulation of TLR2 and TLR4 receptors [156], this could mean that Dexamethasone induces vital NETs. In PE, dexamethasone has been shown to delay neutrophil apoptosis in early-onset preeclampsia; however, it does not affect the rate of neutrophil apoptosis between late-onset preeclampsia and normal pregnancy [157].

Another drug with a potential therapeutic effect, particularly in preeclampsia associated with severe obesity [158], is metformin. This is a hypoglycemic agent that at therapeutic doses has a reduced impact on trophoblast differentiation [159]. Metformin activates AMPK signalling pathways involved in the regulation of NF-κβ/sFlt-1, and Nrf2/HO-1 signalling pathways, thus inhibiting the inflammatory response and oxidative stress [160].

Finally, several systematic reviews have reported that 25-hydroxyvitamin D supplementation before 20 weeks of gestation could reduce NETosis (Table 1) and the risk of PE [161,162], through inhibition of lysosome-associated membrane glycoprotein 3 [163].

## 8. Conclusions

The pathophysiology of preeclampsia is significantly aligned with the activation of neutrophils and the release of mediators, especially those associated with neutrophil extracellular traps (NETs). Consequently, understanding the role of neutrophils and NETs becomes imperative in the context of preeclampsia treatment. Currently, certain drugs are already under review in clinical trials; these include azithromycin, hydroxychloroquine, aspirin, and metformin. Additionally, promising results have already been observed in the use of vitamin D for preeclampsia. This underscores the importance of exploring and considering neutrophil and NET-targeted treatments in the comprehensive approach to managing preeclampsia.

## Data Availability

Data sharing is not applicable.
